# Supplementary material for: Geometric Signatures as Important Factors to Control the Photo-Stabilities of the Phosphorescent Pd(II)/Pt(II) Complexes: A Case Study
Source: Molecules. 2023 Jun 6;28(12):4587. doi: 10.3390/molecules28124587 (PMC10302833; doi:10.3390/molecules28124587)
Supplement: Supplementary file 1 [file molecules-28-04587-s001.zip › molecules-2379795-supplementary.pdf]

Geometric Signatures as Important Factors to Control the Photo-Stabilities of the Phosphorescent Pd(II)/Pt(II) Complexes: A Case Study

Yafei Luo [1,†], Lingkai Tang [2,†], Zhongzhu Chen [1], Zhigang Xu [1], Yanan An [1], Mingyao Li [1], Jianping Hu [2,*] and Dianyong Tang [1,*]

[1] National & Local Joint Engineering Research Center of Targeted and Innovative Therapeutics, Chongqing Engineering Laboratory of Targeted and Innovative Therapeutics, Chongqing Key Laboratory of Kinase Modulators as Innovative Medicine, Chongqing Collaborative Innovation Center of Targeted and Innovative Therapeutics, College of Pharmacy (International Academy of Targeted Therapeutics and Innovation), Chongqing University of Arts and Sciences, Chongqing 402160, China

[2] Key Laboratory of Medicinal and Edible Plants Resources Development of Sichuan Education Department, School of Pharmacy, Chengdu University, Chengdu 610106, China

* Correspondence: hujianping@cdu.edu.cn (J.H.); tdy@cqwu.edu.cn (D.T.)

† These authors contributed equally to this work.

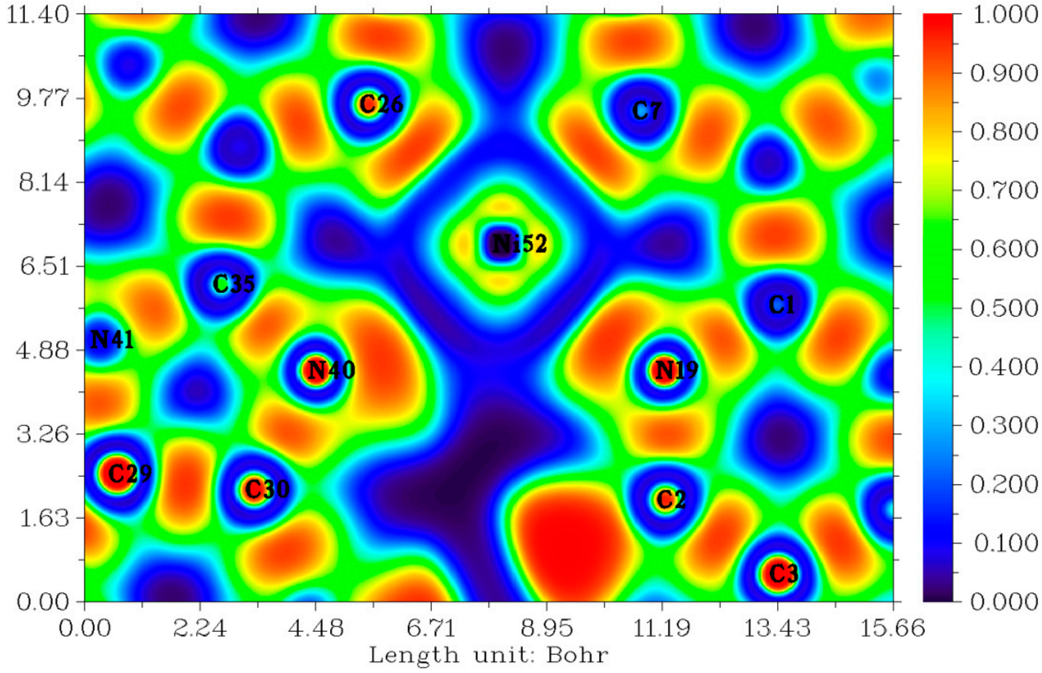

Figure S1. Two-dimensional color-filled maps of ELF for **Ni-1** at the T_1 state.

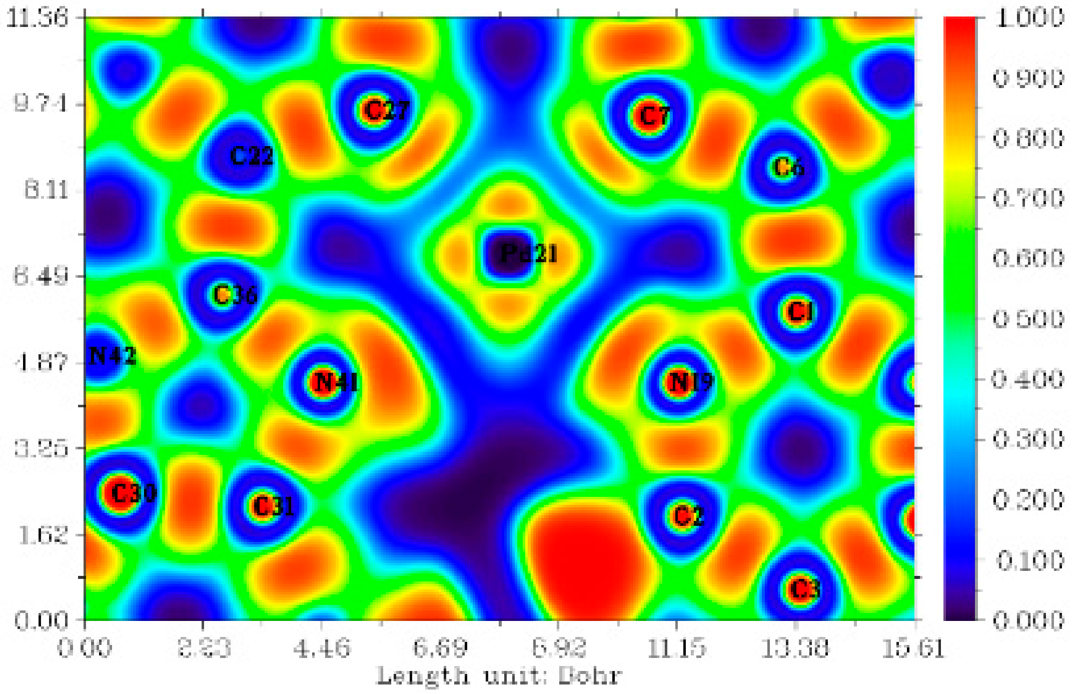

Figure S2. Two-dimensional color-filled maps of ELF for **Pd-1** at the T_1 state.

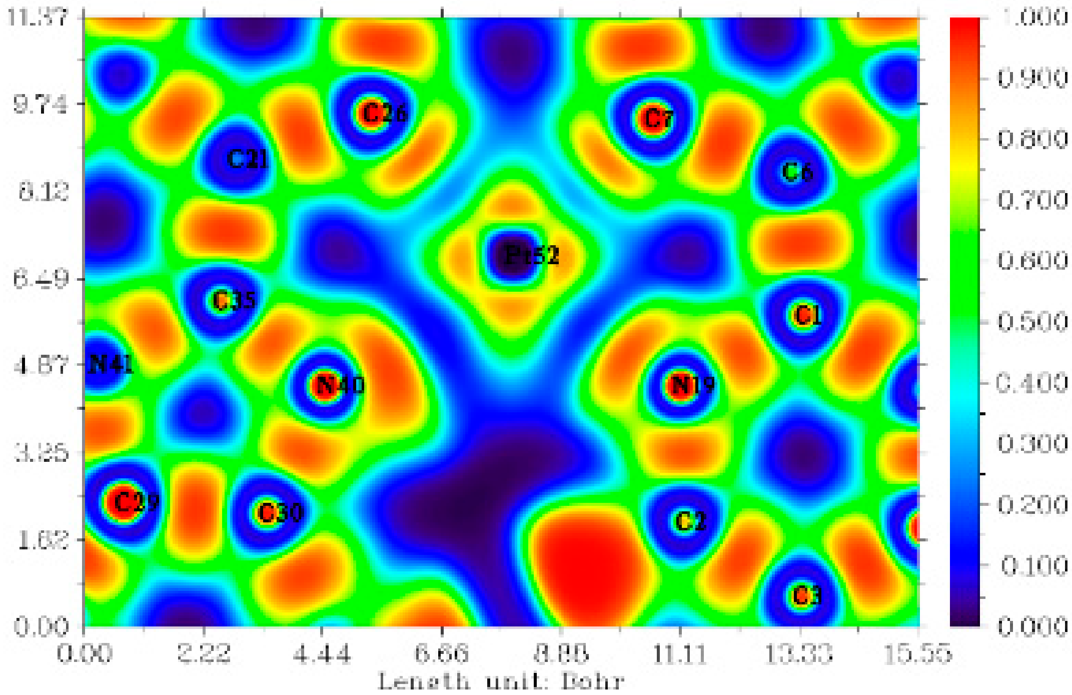

Figure S3. Two-dimensional color-filled maps of ELF for **Pt-1** at the T_1 state.

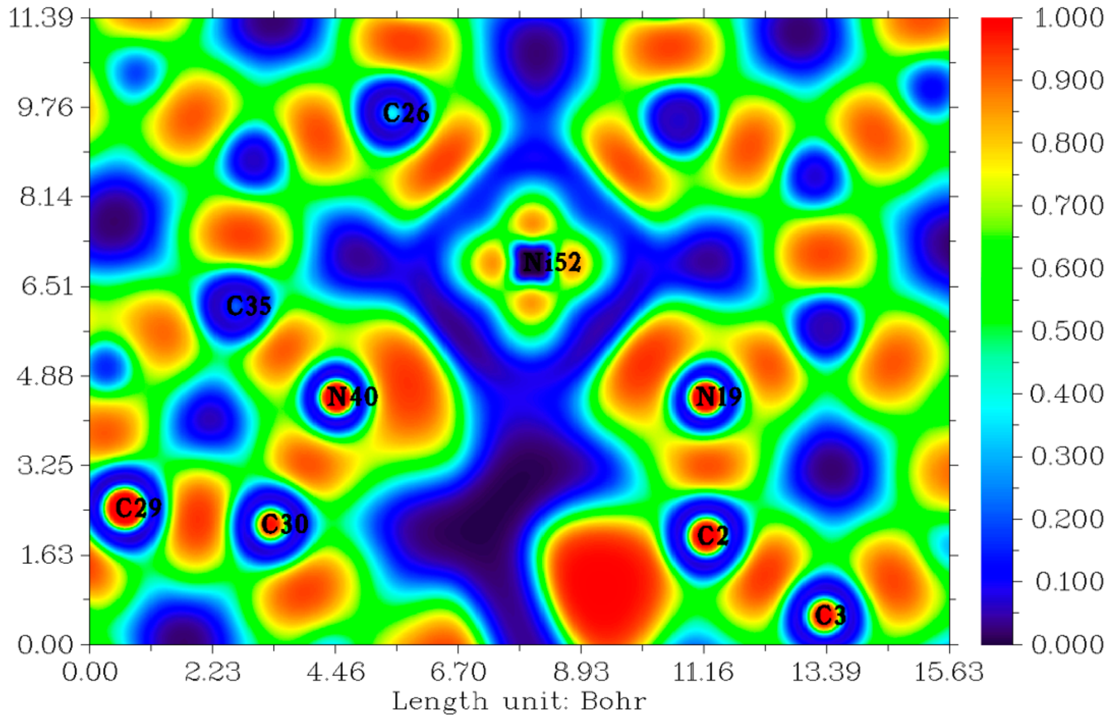

Figure S4. Two-dimensional color-filled maps of ELF for **Ni-1** at the S_1 state.

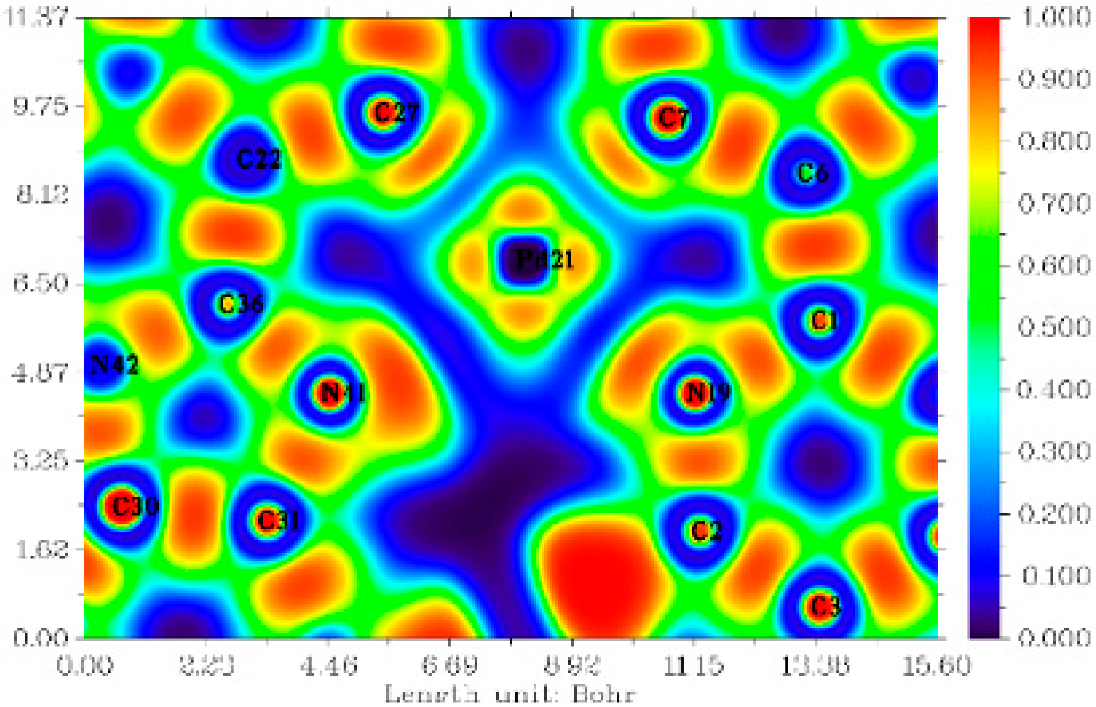

Figure S5. Two-dimensional color-filled maps of ELF for **Pd-1** at the S_1 state.

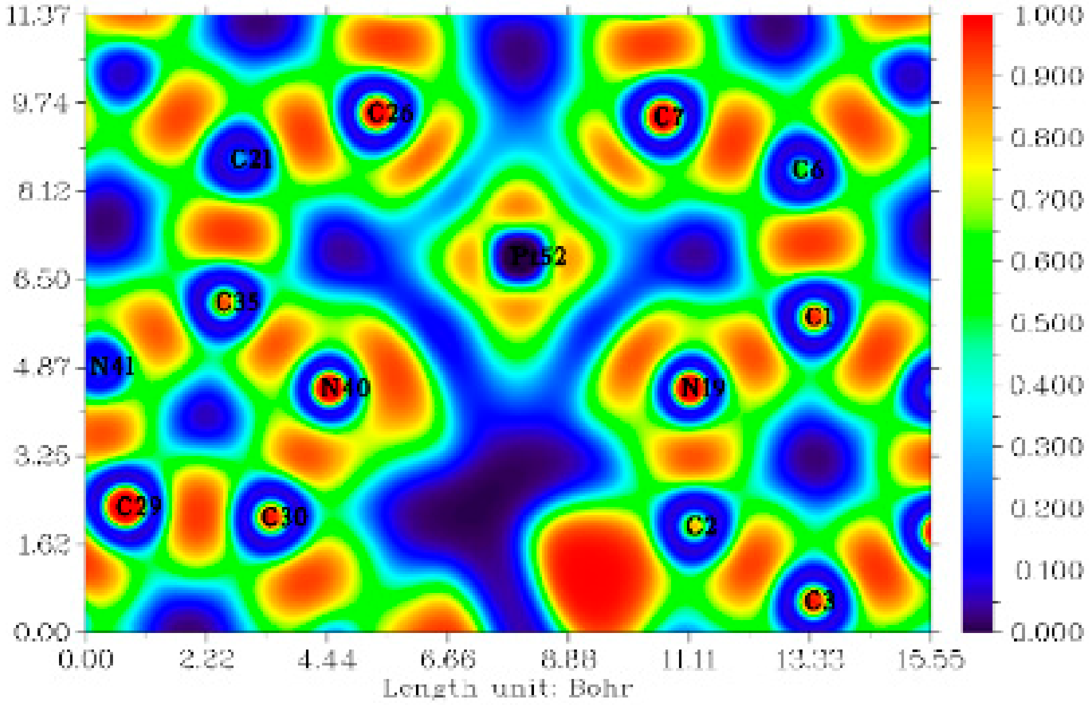

Figure S6. Two-dimensional color-filled maps of ELF for **Pt-1** at the S_1 state.

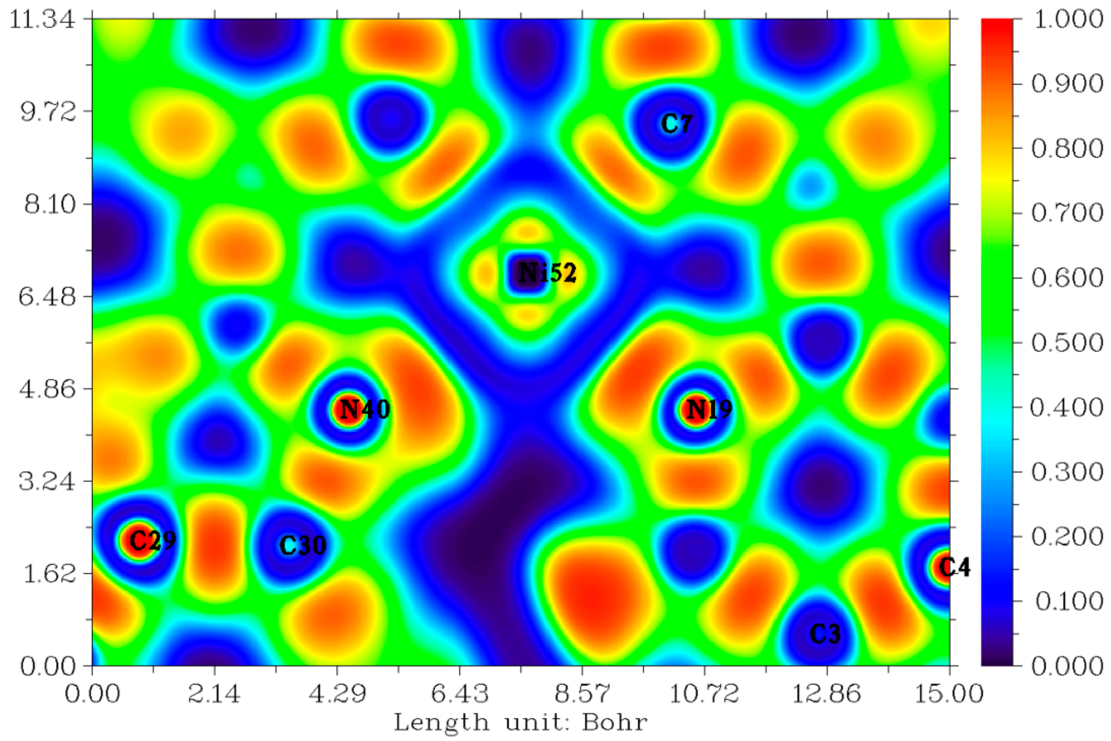

Figure S7. Two-dimensional color-filled maps of ELF for **Ni-1** at the cation state.

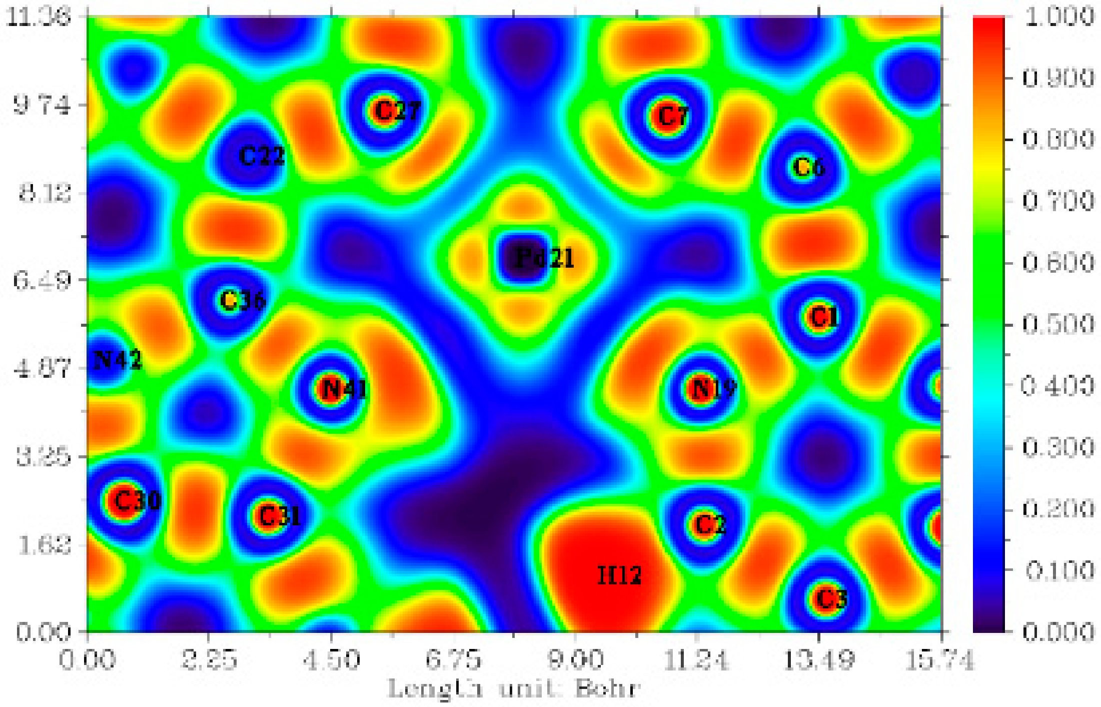

Figure S8. Two-dimensional color-filled maps of ELF for **Pd-1** at the cation state.

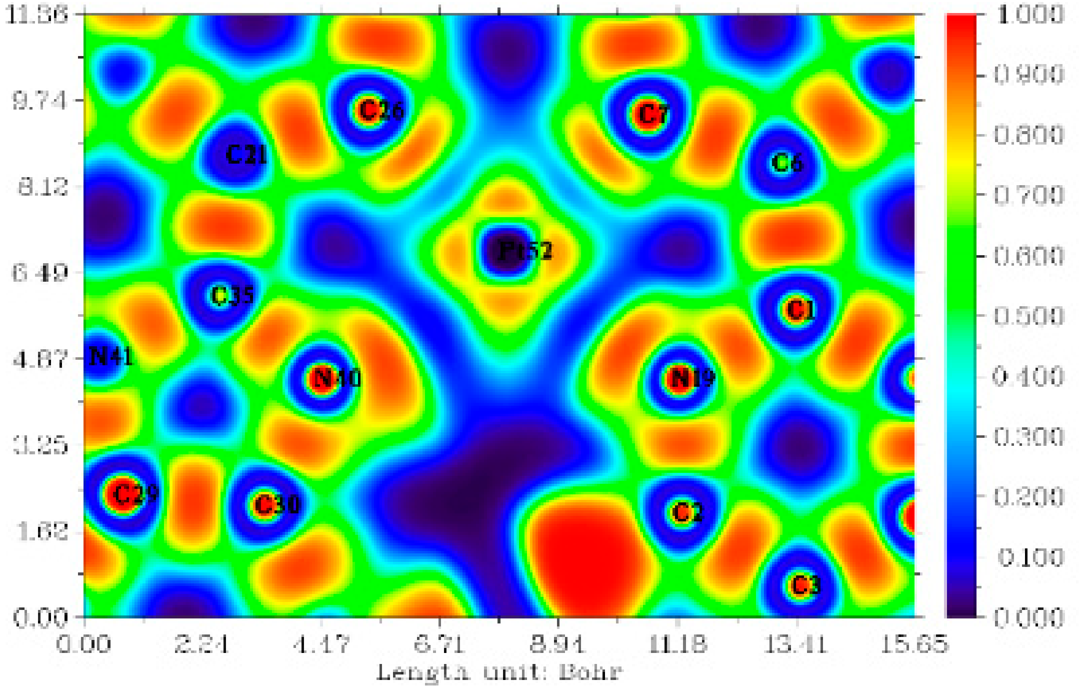

Figure S9. Two-dimensional color-filled maps of ELF for **Pt-1** at the cation state.

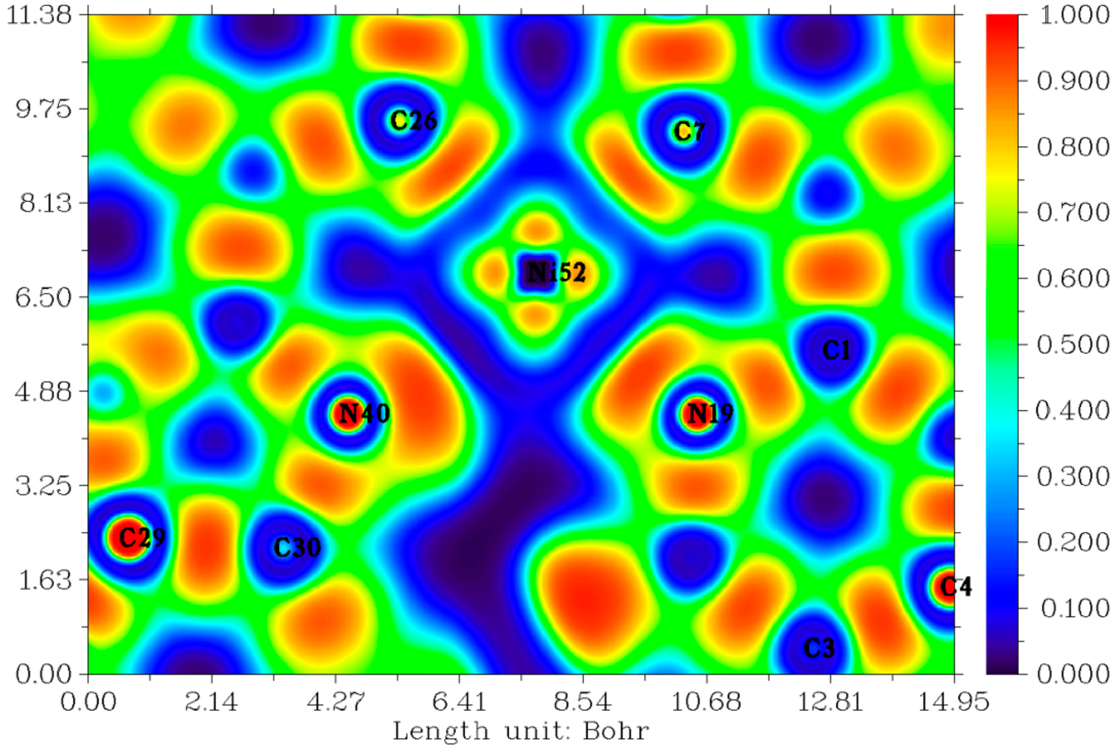

Figure S10. Two-dimensional color-filled maps of ELF for **Ni-1** at the anion state.

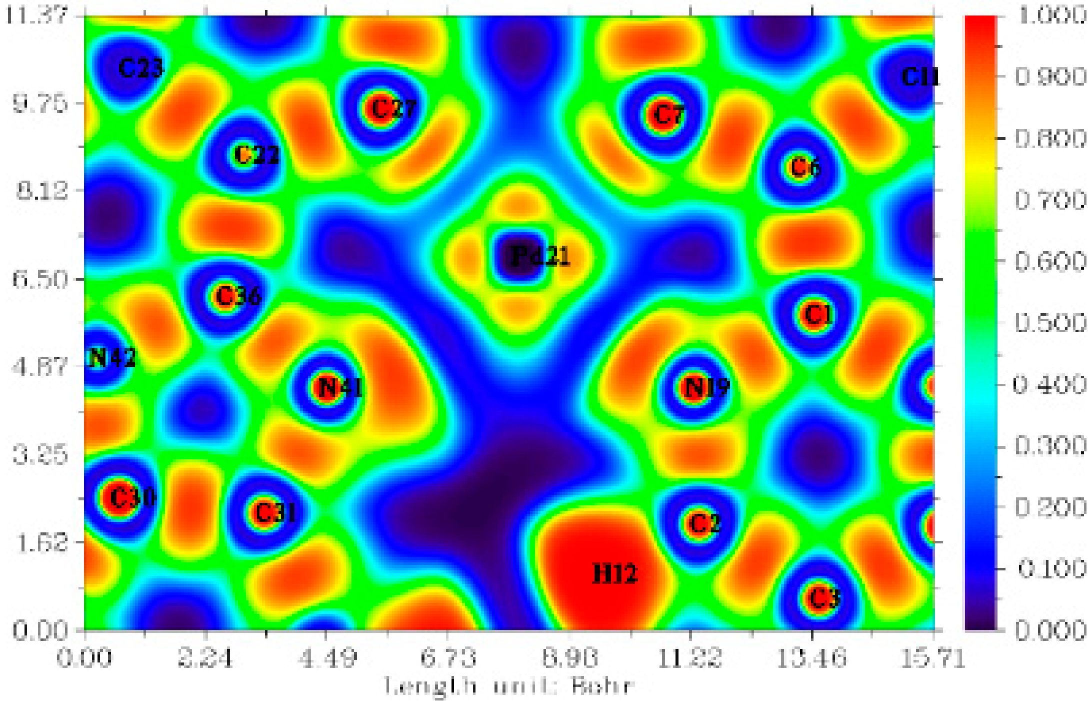

Figure S11. Two-dimensional color-filled maps of ELF for **Pd-1** at the anion state.

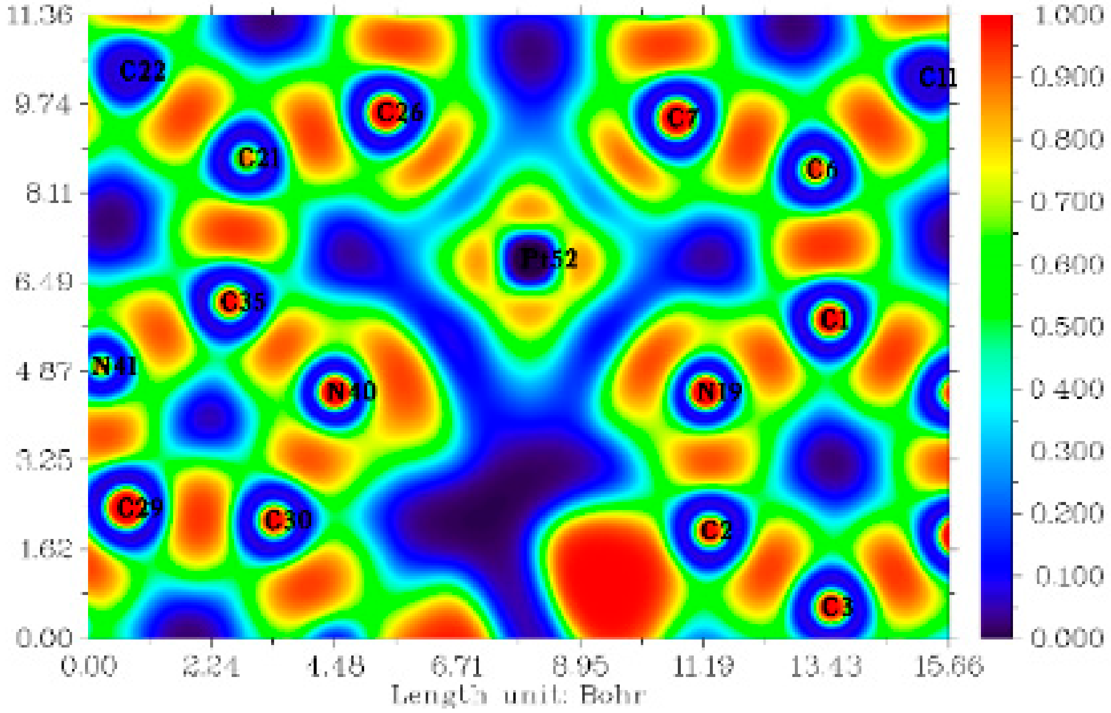

Figure S12. Two-dimensional color-filled maps of ELF for **Pt-1** at the anion state.

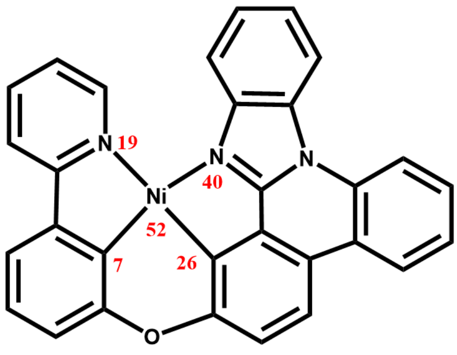

Ni-1

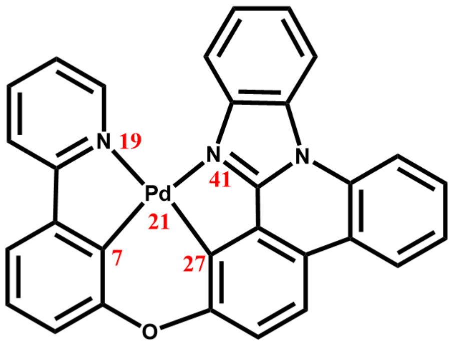

Pd-1

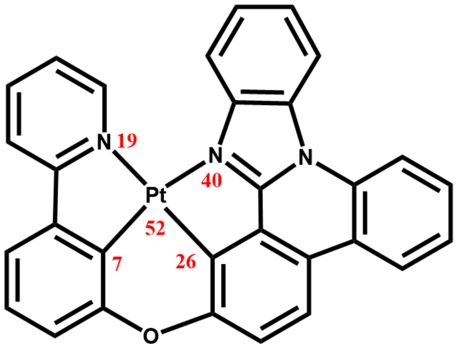

Pt-1

Figure S13. Chemical geometries of **Ni-1**, **Pd-1** and **Pt-1** with key labels, respectively.

Table S1. Topological parameters of the electron density calculated at the bond critical point (BCP) (3, -1) of Pd/Pt-N and Pd/Pt-C bonds for **Ni-1**, **Pd-1** and **Pt-1** at the S_0, T_1, S_1, + and - states, respectively.

Ni-1

	S_0			T_1			S_1			+			-		
	ρ_{BCP}	$\nabla^2\rho_{BCP}$	E_{BCP}	ρ_{BCP}	$\nabla^2\rho_{BCP}$	E_{BCP}	ρ_{BCP}	$\nabla^2\rho_{BCP}$	E_{BCP}	ρ_{BCP}	$\nabla^2\rho_{BCP}$	E_{BCP}	ρ_{BCP}	$\nabla^2\rho_{BCP}$	E_{BCP}
Ni52-N40	0.055	0.327	0.001	0.041	0.211	0.003	0.039	0.221	0.004	0.067	0.362	-0.003	0.053	0.318	0.002
Ni52-C26	0.128	0.280	-0.048	0.102	0.329	-0.027	0.104	0.238	-0.028	0.115	0.207	-0.036	0.129	0.288	-0.048
Ni52-C7	0.131	0.262	-0.049	0.099	0.321	-0.024	0.102	0.224	-0.025	0.115	0.198	-0.035	0.131	0.263	-0.049
Ni52-N19	0.084	0.483	-0.013	0.063	0.363	-0.0009	0.061	0.356	-0.0008	0.083	0.439	-0.011	0.091	0.505	-0.017

Pd-1

	S_0			T_1			S_1			+			-		
	ρ_{BCP}	$\nabla^2\rho_{BCP}$	E_{BCP}	ρ_{BCP}	$\nabla^2\rho_{BCP}$	E_{BCP}	ρ_{BCP}	$\nabla^2\rho_{BCP}$	E_{BCP}	ρ_{BCP}	$\nabla^2\rho_{BCP}$	E_{BCP}	ρ_{BCP}	$\nabla^2\rho_{BCP}$	E_{BCP}
Pd21-N41	0.052	0.222	-0.007	0.064	0.266	-0.012	0.059	0.248	-0.010	0.060	0.249	-0.010	0.058	0.247	-0.009
Pd21-C27	0.135	0.217	-0.061	0.142	0.250	-0.066	0.138	0.263	-0.062	0.139	0.247	-0.063	0.136	0.229	-0.061
Pd21-C7	0.139	0.198	-0.064	0.143	0.209	-0.067	0.144	0.182	-0.070	0.141	0.206	-0.066	0.140	0.203	-0.065
Pd21-N19	0.075	0.324	-0.016	0.087	0.364	-0.021	0.094	0.375	-0.025	0.081	0.341	-0.019	0.083	0.363	-0.019

Pt-1

	S_0			T_1			S_1			+			-		
	ρ_{BCP}	$\nabla^2\rho_{BCP}$	E_{BCP}	ρ_{BCP}	$\nabla^2\rho_{BCP}$	E_{BCP}	ρ_{BCP}	$\nabla^2\rho_{BCP}$	E_{BCP}	ρ_{BCP}	$\nabla^2\rho_{BCP}$	E_{BCP}	ρ_{BCP}	$\nabla^2\rho_{BCP}$	E_{BCP}
Pt52-N40	0.067	0.273	-0.011	0.071	0.280	-0.013	0.071	0.284	-0.013	0.073	0.286	-0.013	0.067	0.272	-0.011
Pt52-C26	0.153	0.246	-0.068	0.153	0.256	-0.067	0.155	0.268	-0.069	0.158	0.254	-0.072	0.153	0.259	-0.067
Pt52-C7	0.158	0.218	-0.073	0.169	0.215	-0.083	0.163	0.194	-0.078	0.161	0.220	-0.075	0.158	0.217	-0.073
Pt52-N19	0.089	0.378	-0.019	0.104	0.417	-0.026	0.104	0.404	-0.026	0.092	0.371	-0.021	0.095	0.402	-0.022

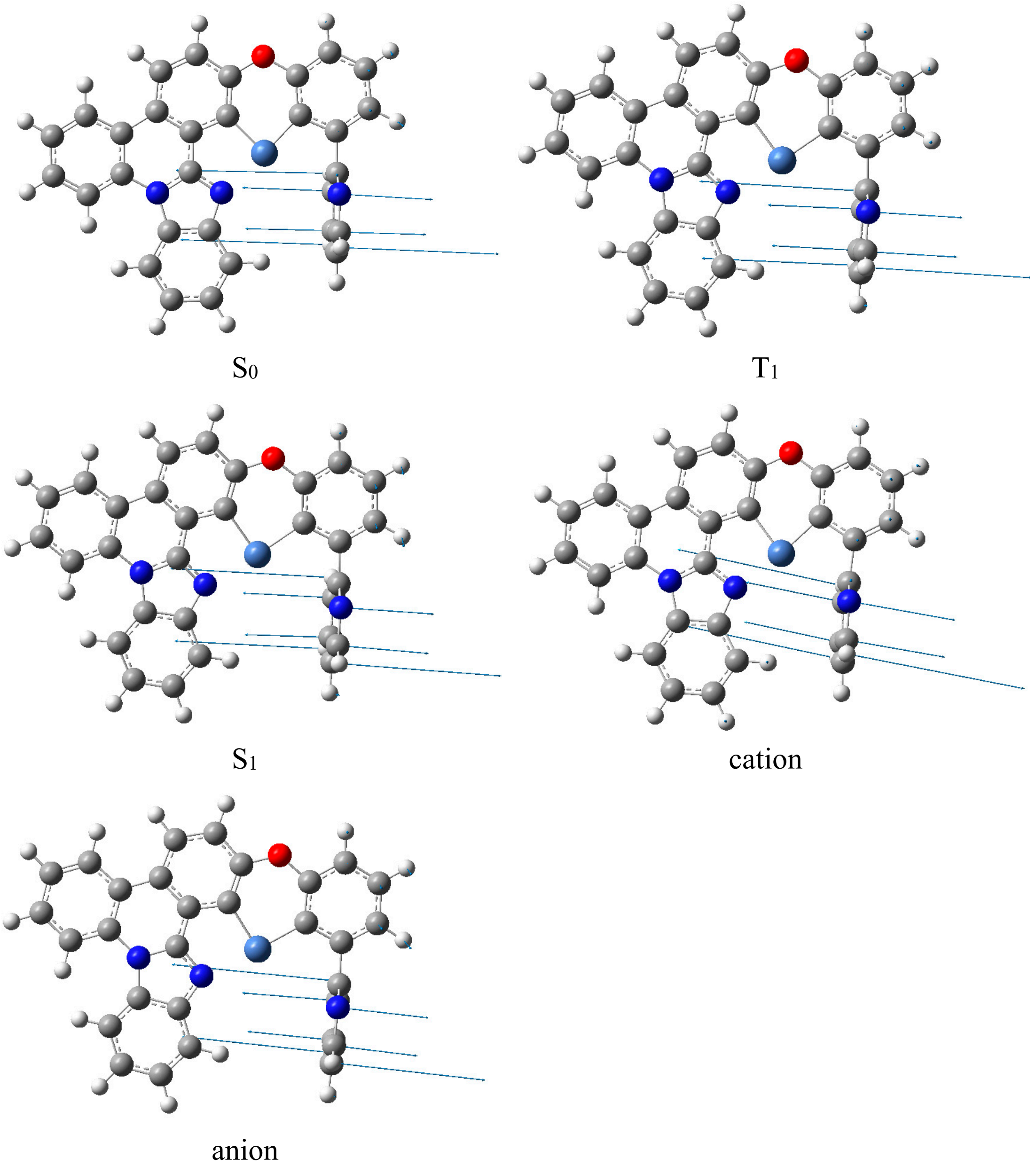

Figure S14. Vibration forms of TS for **Ni-1** at the S_0, T_1, S_1, cation and anion states, respectively.

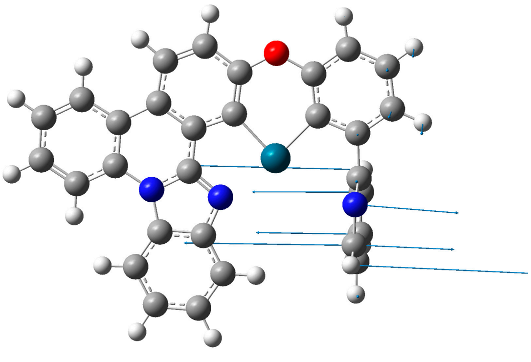

S_0

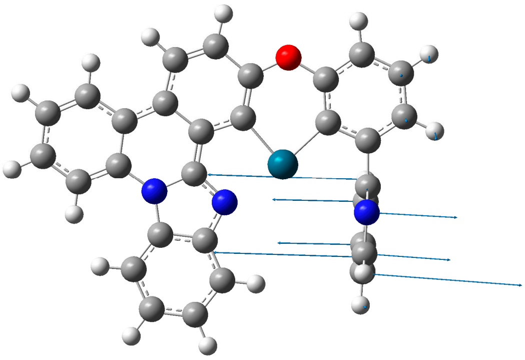

T_1

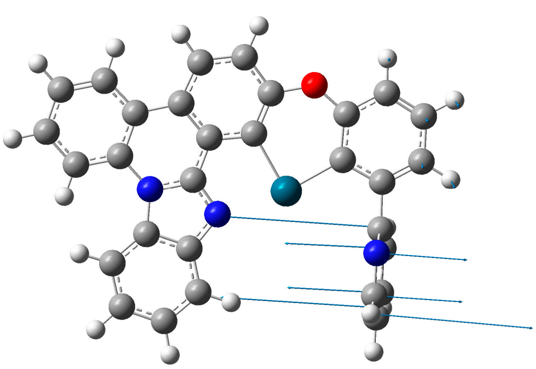

S_1

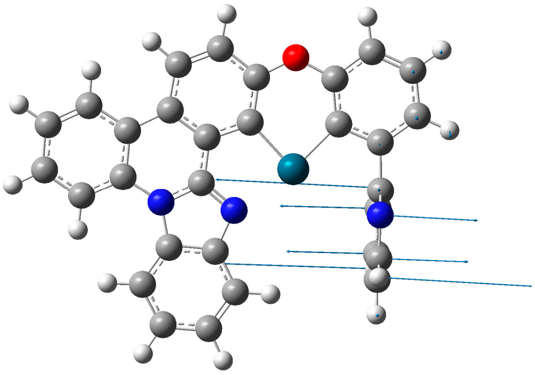

cation

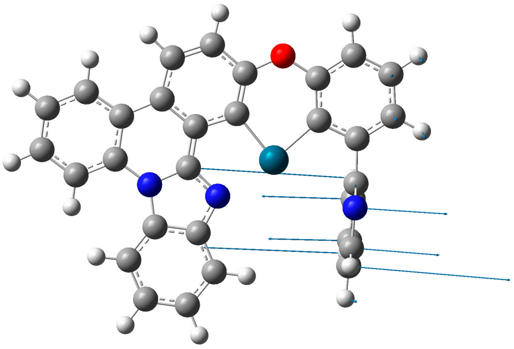

anion

Figure S15. Vibration forms of TS for **Pd-1** at the S_0, T_1, S_1, cation and anion states, respectively.

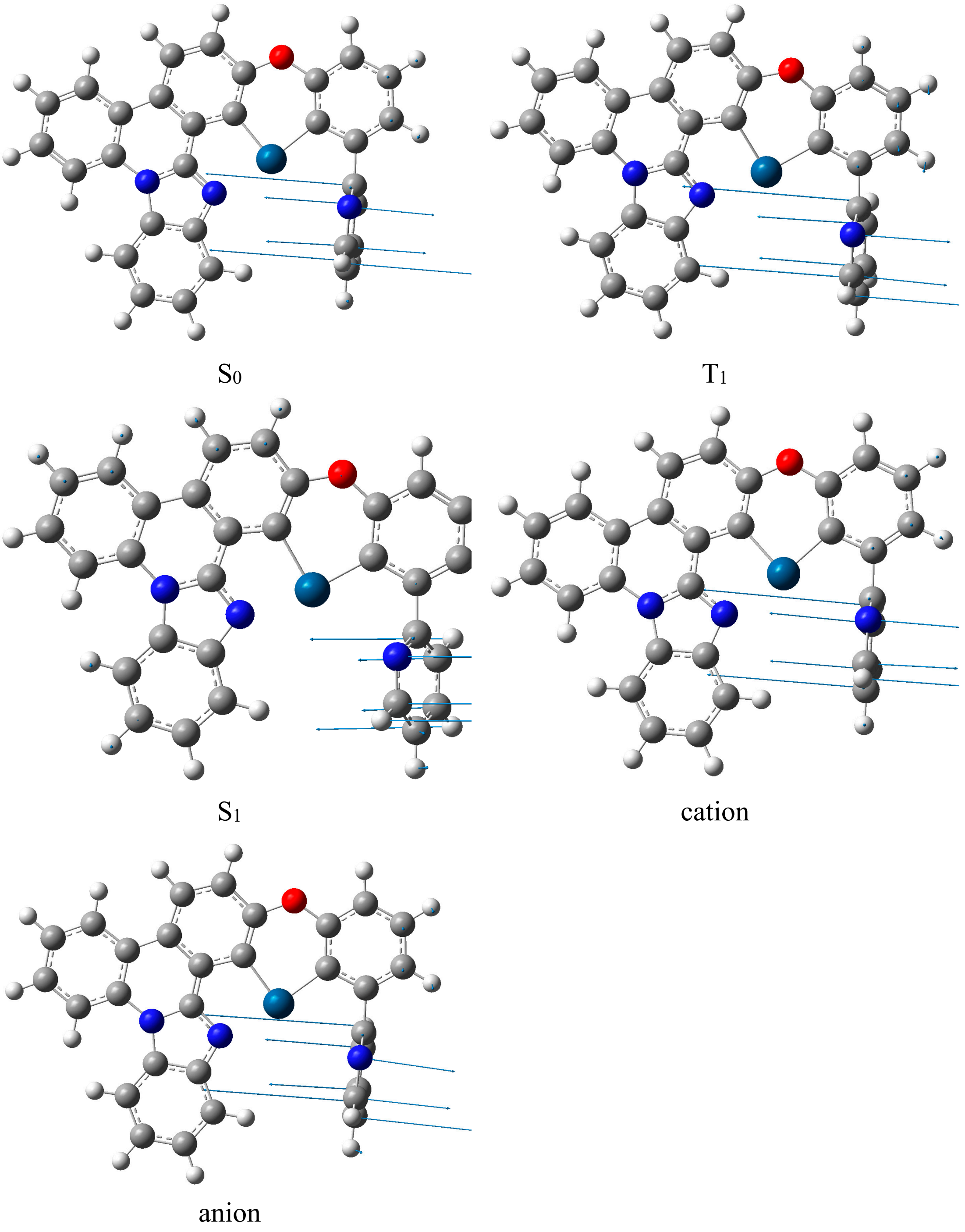

Figure S16. Vibration forms of TS for **Pt-1** at the S_0, T_1, S_1, cation and anion states, respectively.

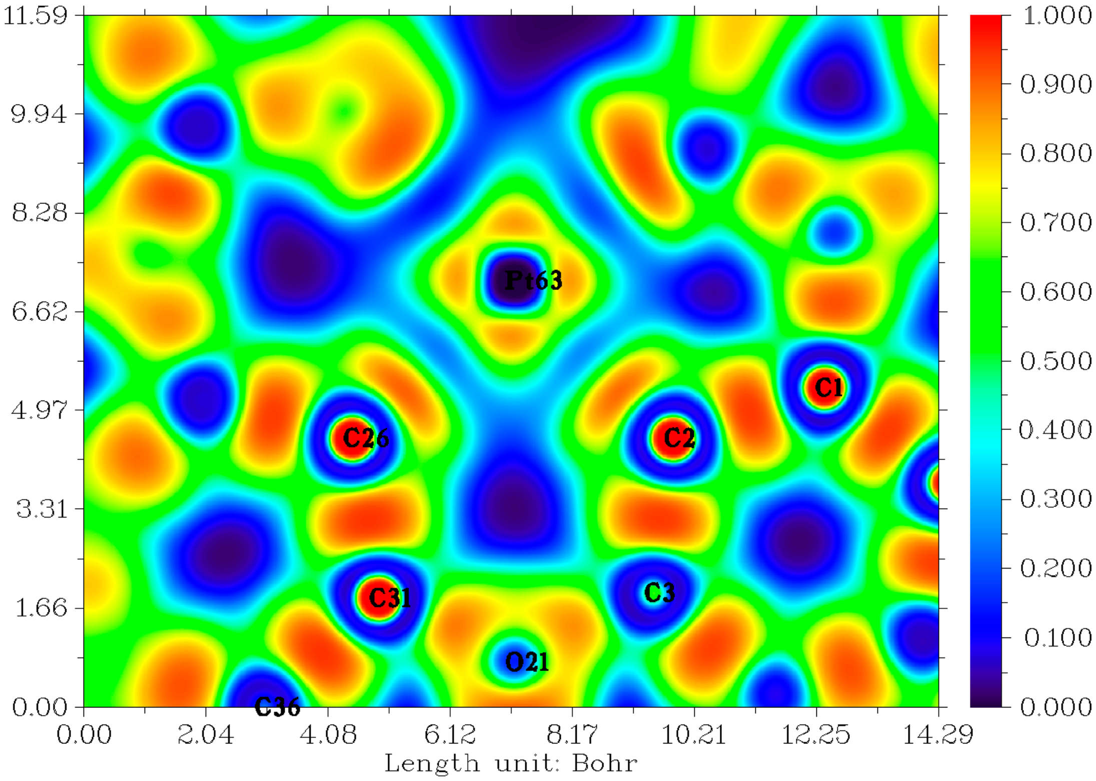

Figure S17. Two-dimensional color-filled maps of ELF for **Pt-2** at the T_1 state.

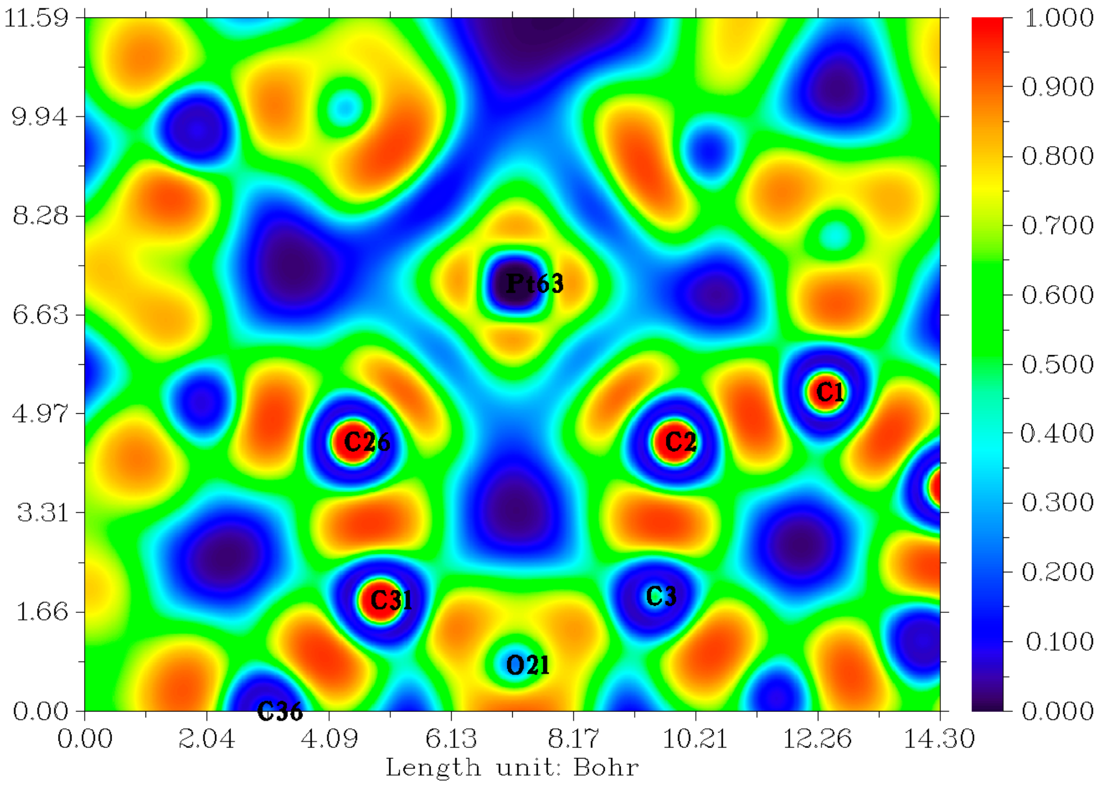

Figure S18. Two-dimensional color-filled maps of ELF for **Pt-2** at the S_1 state.

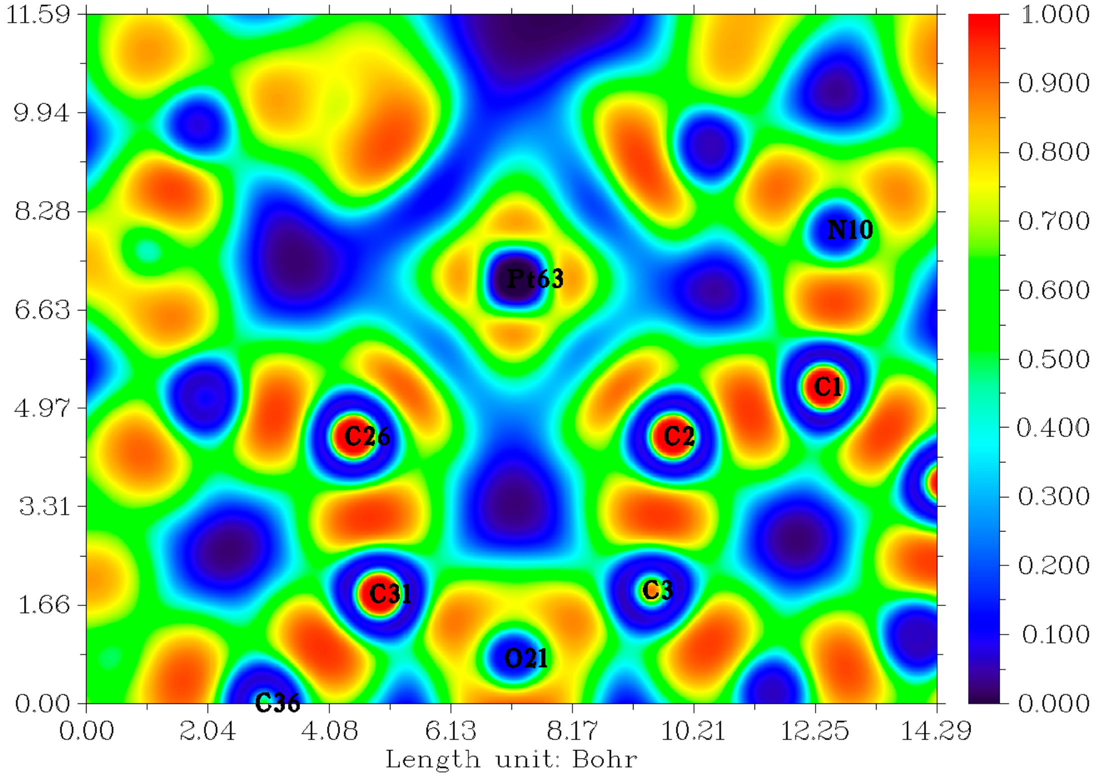

Figure S19. Two-dimensional color-filled maps of ELF for **Pt-2** at the cation state.

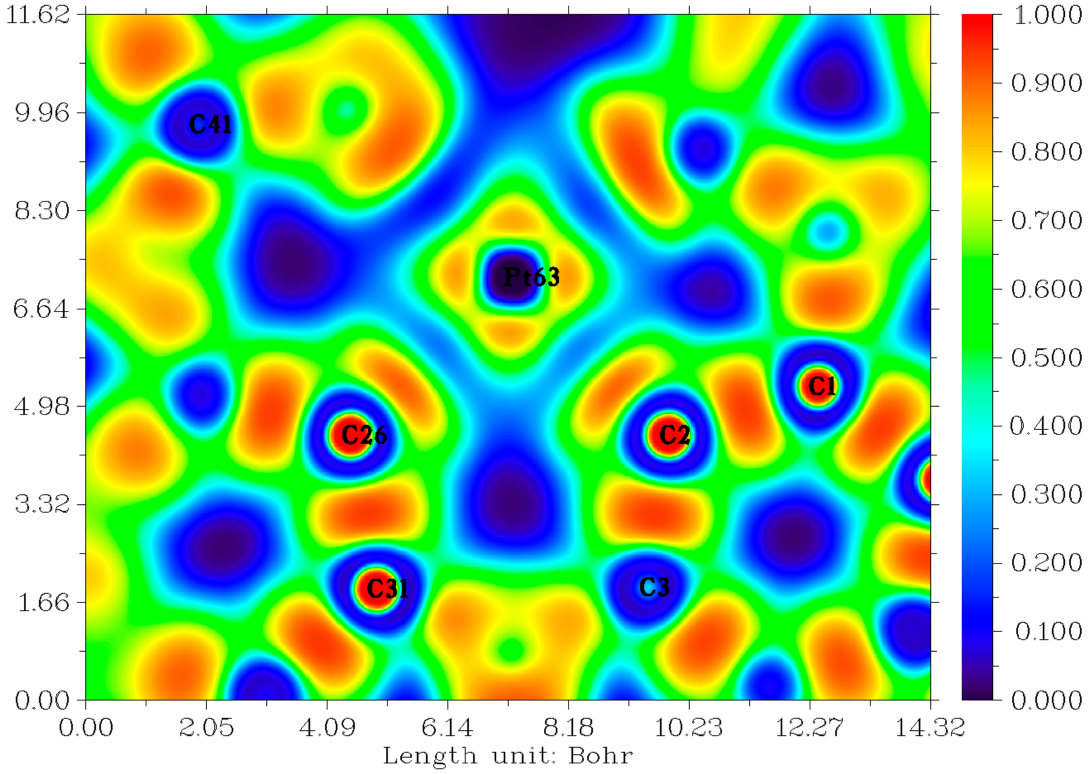

Figure S20. Two-dimensional color-filled maps of ELF for **Pt-2** at the anion state.

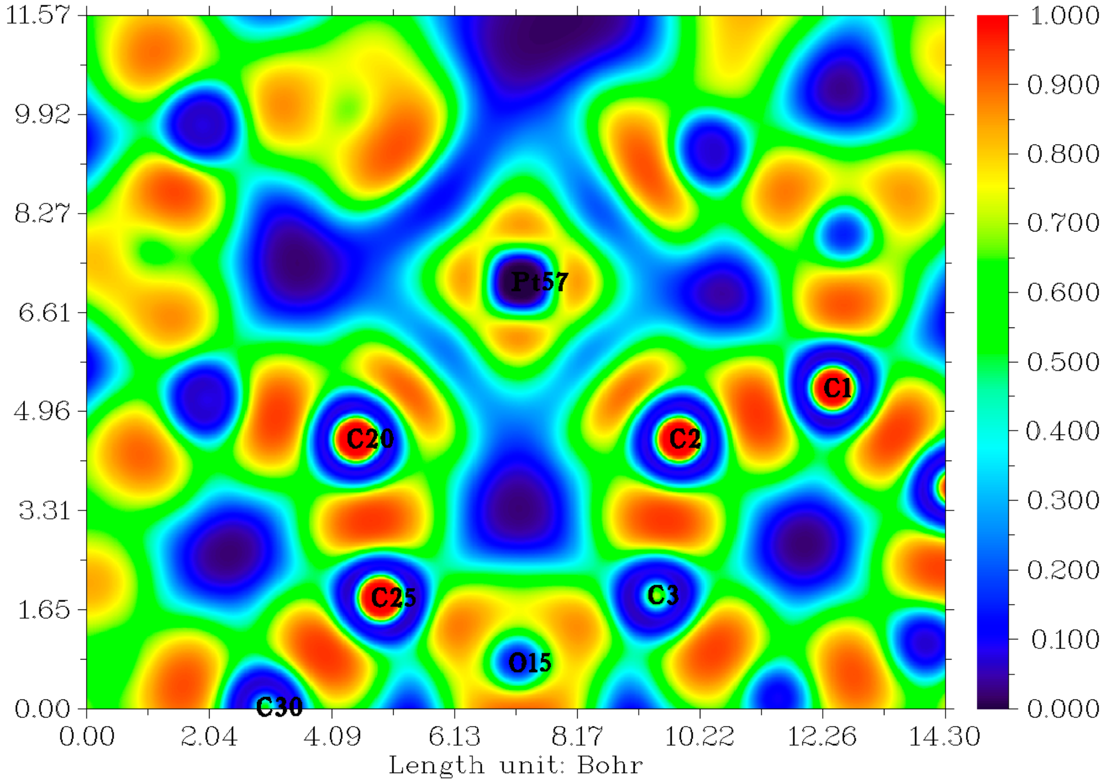

Figure S21. Two-dimensional color-filled maps of ELF for **Pt-3** at the T_1 state.

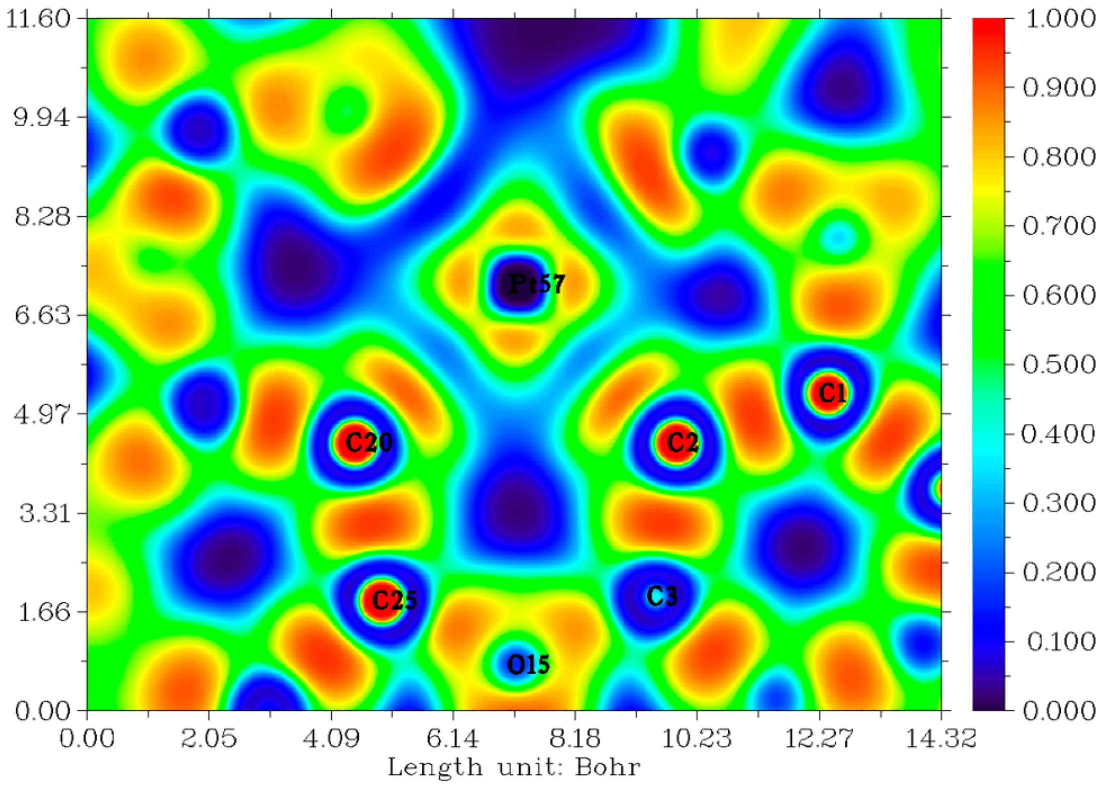

Figure S22. Two-dimensional color-filled maps of ELF for **Pt-3** at the S_1 state.

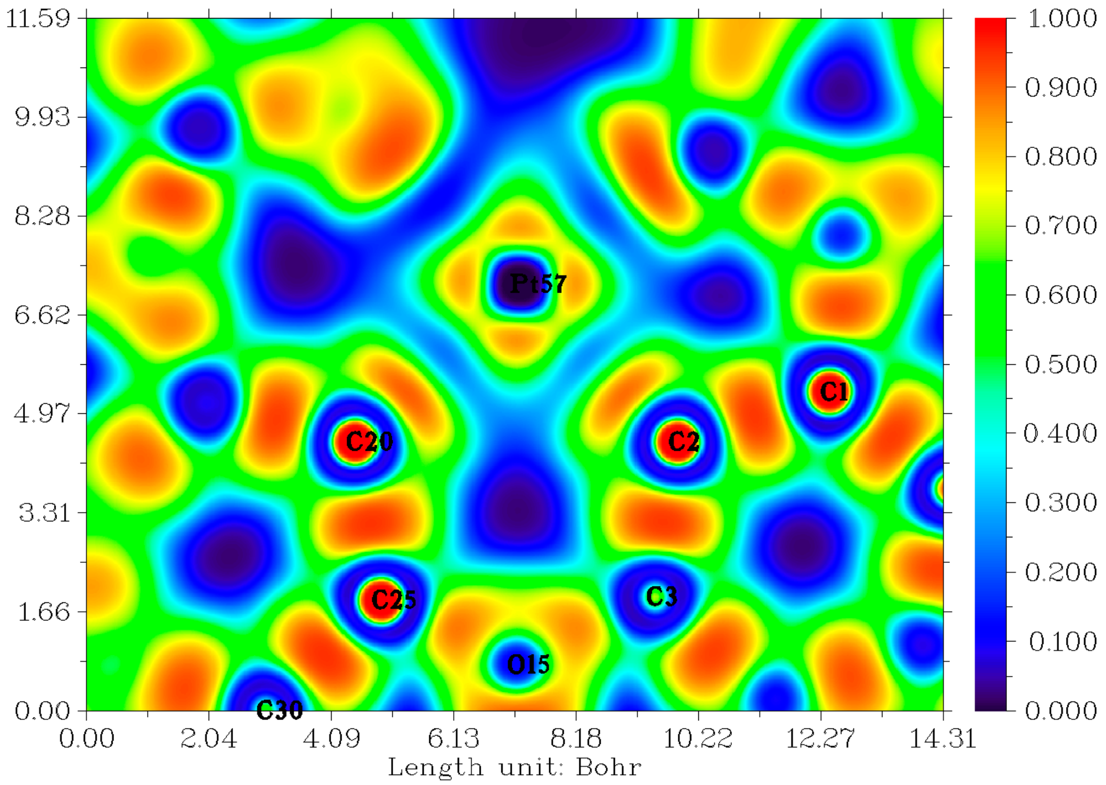

Figure S23. Two-dimensional color-filled maps of ELF for **Pt-3** at the cation state.

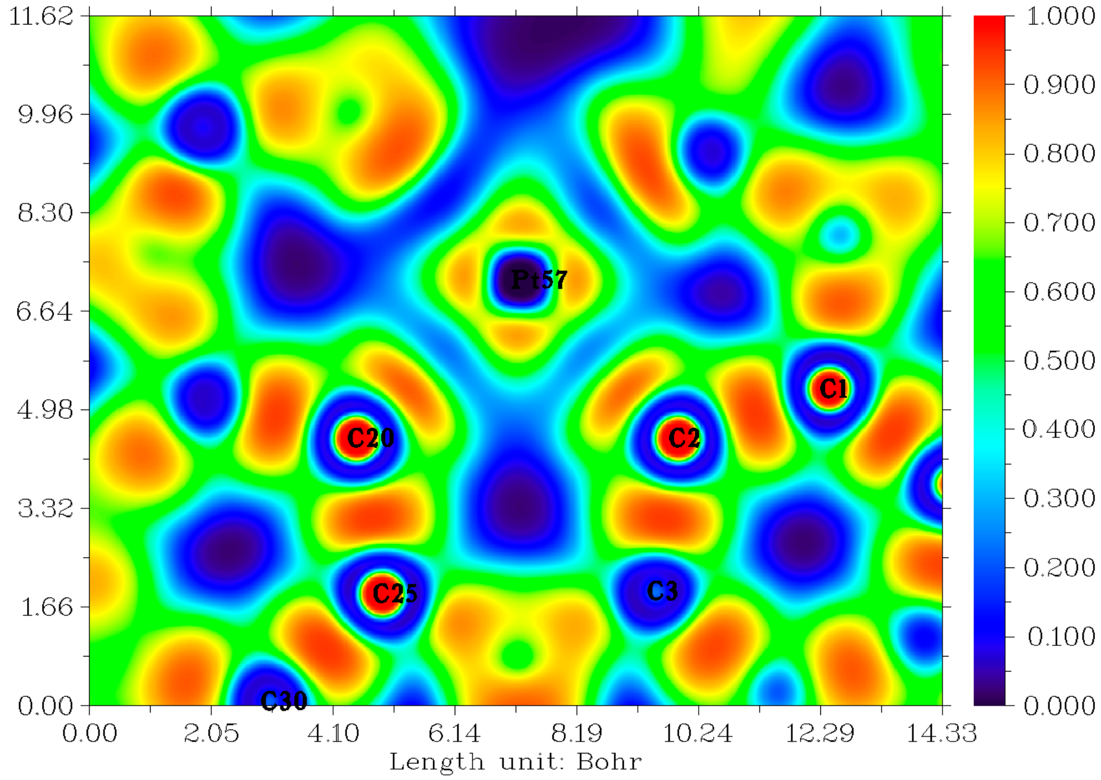

Figure S24. Two-dimensional color-filled maps of ELF for **Pt-3** at the anion state.

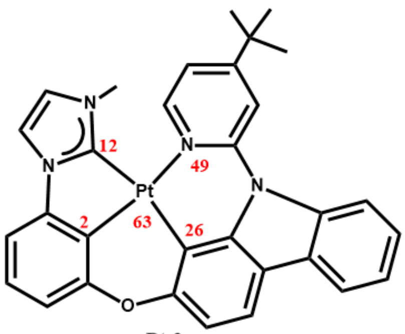

Pt-2

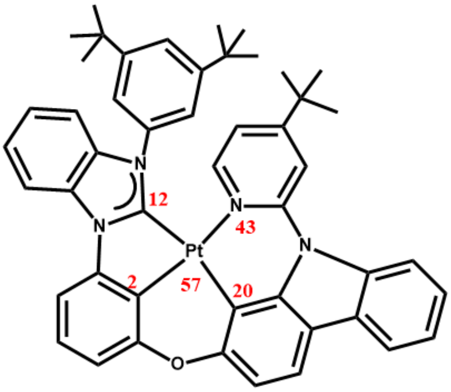

Pt-3

Figure S25. Chemical geometries of **Pt-2** and **Pt-3** with key labels.

Table S2. Topological parameters of the electron density calculated at the bond critical point (BCP) (3, -1) of Pd/Pt-N and Pd/Pt-C bonds for **Pt-2** and **Pt-3** at the S_0, T_1, S_1, + and - states, respectively.

**Pt-2**															
		S_0			T_1			S_1			+			-	
	ρ_{BCP}	$\nabla^2\rho_{BCP}$	E_{BCP}	ρ_{BCP}	$\nabla^2\rho_{BCP}$	E_{BCP}	ρ_{BCP}	$\nabla^2\rho_{BCP}$	E_{BCP}	ρ_{BCP}	$\nabla^2\rho_{BCP}$	E_{BCP}	ρ_{BCP}	$\nabla^2\rho_{BCP}$	E_{BCP}
**Pt63-C2**	0.148	0.218	-0.065	0.150	0.244	-0.065	0.149	0.222	-0.065	0.152	0.229	-0.068	0.146	0.251	-0.062
**Pt63-C12**	0.119	0.332	-0.042	0.124	0.326	-0.044	0.121	0.339	-0.042	0.119	0.312	-0.041	0.125	0.350	-0.045
**Pt63-C26**	0.136	0.252	-0.055	0.143	0.285	-0.059	0.142	0.260	-0.059	0.140	0.274	-0.057	0.136	0.257	-0.054
**Pt63-N49**	0.083	0.358	-0.016	0.094	0.380	-0.022	0.085	0.376	-0.017	0.084	0.348	-0.017	0.094	0.381	-0.021
**Pt-3**															
		S_0			T_1			S_1			+			-	
	ρ_{BCP}	$\nabla^2\rho_{BCP}$	E_{BCP}	ρ_{BCP}	$\nabla^2\rho_{BCP}$	E_{BCP}	ρ_{BCP}	$\nabla^2\rho_{BCP}$	E_{BCP}	ρ_{BCP}	$\nabla^2\rho_{BCP}$	E_{BCP}	ρ_{BCP}	$\nabla^2\rho_{BCP}$	E_{BCP}
**Pt57-C2**	0.149	0.215	-0.066	0.155	0.238	-0.070	0.150	0.220	-0.066	0.153	0.229	-0.068	0.147	0.233	-0.064
**Pt57-C12**	0.125	0.351	-0.044	0.129	0.326	-0.048	0.123	0.346	-0.043	0.123	0.325	-0.044	0.128	0.348	-0.047
**Pt57-C20**	0.136	0.250	-0.055	0.140	0.283	-0.057	0.139	0.252	-0.057	0.139	0.275	-0.057	0.134	0.260	-0.053
**Pt57-N43**	0.083	0.354	-0.016	0.091	0.376	-0.020	0.086	0.380	-0.017	0.084	0.348	-0.017	0.092	0.389	-0.020

Table S3. Laplacian bond orders of Pt-N and Pt-C bonds of **Pt-2** and **Pt-3**.

			**Pt-2**		
	S_0	T_1	S_1	+	-
Pt63-C2	0.58	0.61	0.59	0.61	0.60
Pt63-C12	0.58	0.60	0.60	0.57	0.62
Pt63-C26	0.57	0.63	0.60	0.60	0.57
Pt63-N49	0.21	0.23	0.23	0.20	0.23
			**Pt-3**		
	S_0	T_1	S_1	+	-
Pt57-C2	0.58	0.63	0.59	0.61	0.59
Pt57-C12	0.64	0.63	0.63	0.62	0.65
Pt57-C20	0.57	0.61	0.58	0.60	0.57
Pt57-N43	0.20	0.22	0.23	0.20	0.23

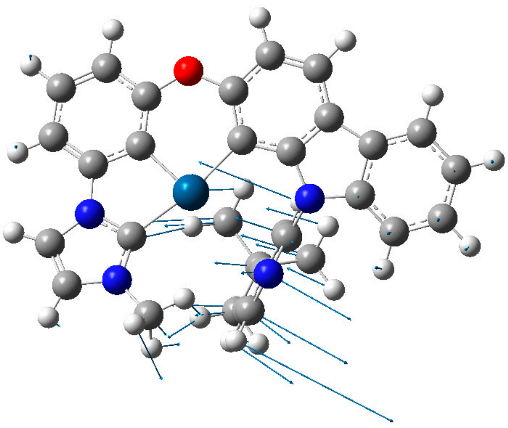

S0

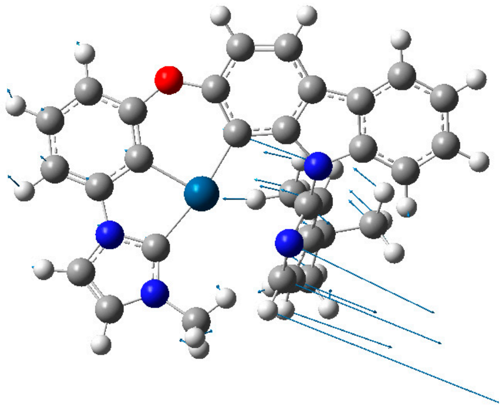

T1

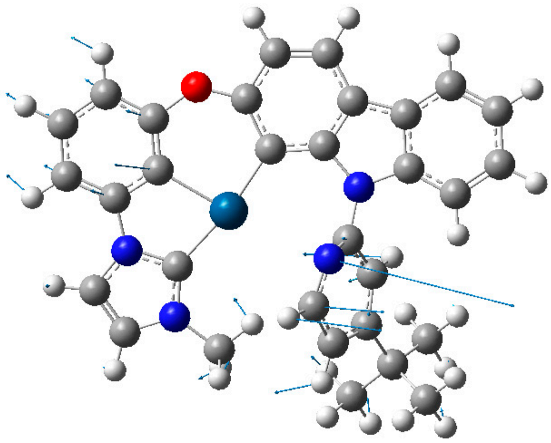

S1

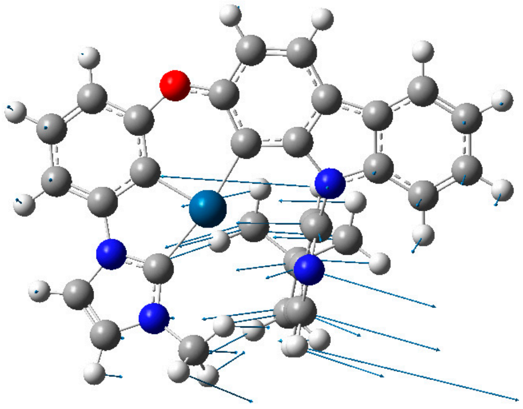

cation

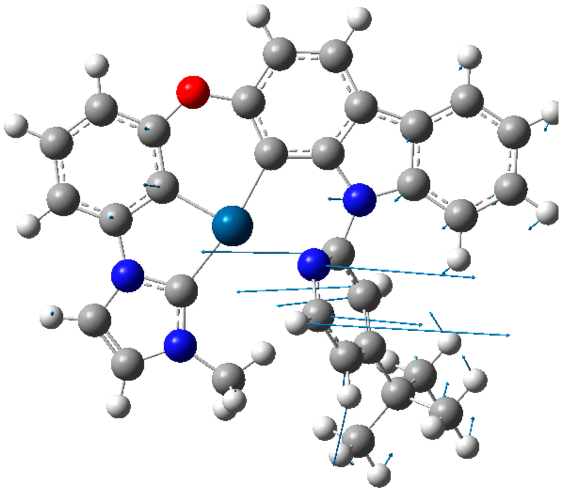

anion

Figure S26. Vibration forms for **Pt-2** at the S_0, T_1, S_1, cation and anion states, respectively.

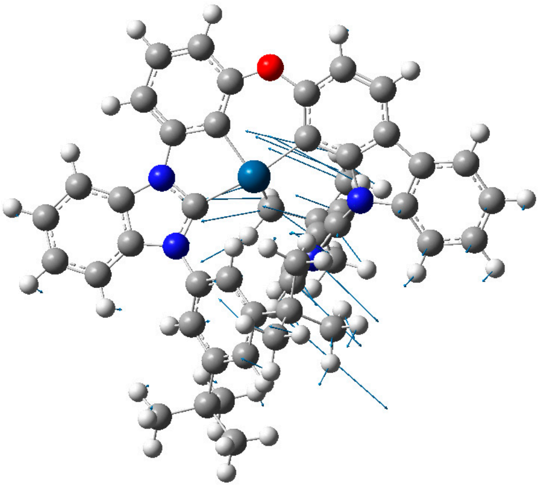

S_0

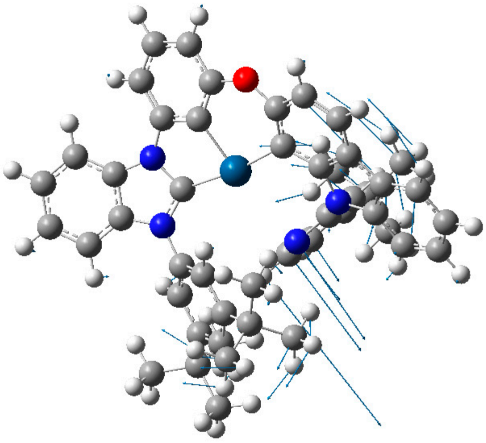

T_1

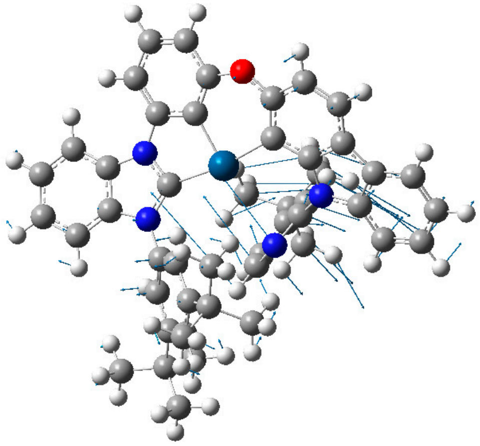

cation

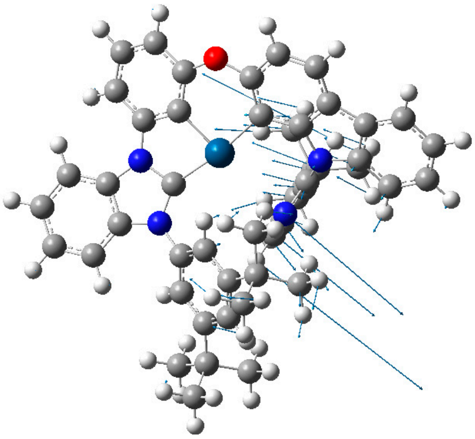

anion

Figure S27. Vibration forms for **Pt-3** at the S_0, T_1, S_1, cation and anion states, respectively.

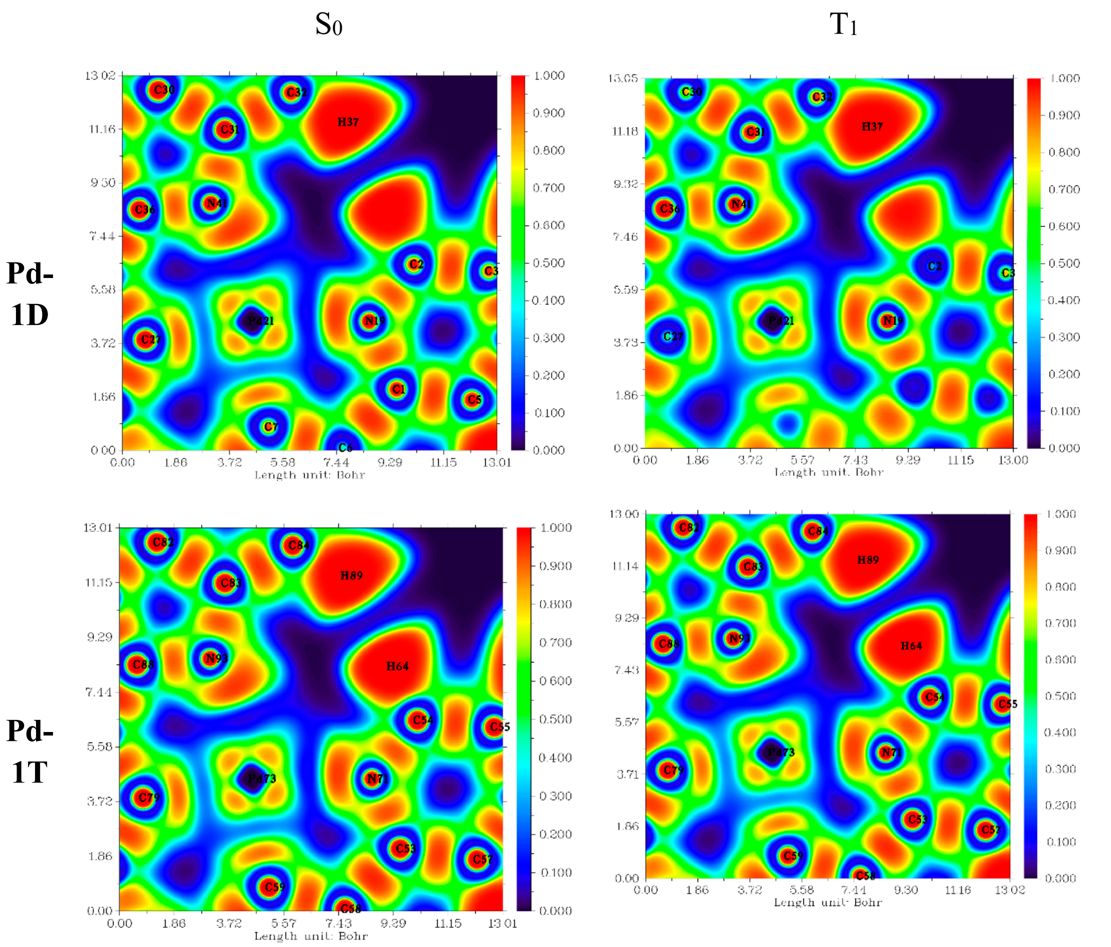

Figure S28. Two-dimensional color-filled maps of ELF for **Pd-1D** and **Pd-1T** at the S_0 and T_1 states, respectively.

Table S4. Topological parameters of the electron density calculated at the bond critical point (BCP) (3, -1) of Pd/Pt-N and Pd/Pt-C bonds for **Pd-1D** and **Pd-1T** at the S_0, T_1 states.

	**Pd-1D**					
		S_0			T_1	
	ρ_{BCP}	$\nabla^2\rho_{BCP}$	E_{BCP}	ρ_{BCP}	$\nabla^2\rho_{BCP}$	E_{BCP}
Pd21-C7	0.139	0.202	-0.065	0.140	0.156	-0.067
Pd21-N19	0.079	0.345	-0.018	0.082	0.341	-0.019
Pd21-C27	0.136	0.226	-0.061	0.137	0.180	-0.064
Pd21-N41	0.057	0.244	-0.009	0.059	0.244	-0.010
	**Pd-1T**					
		S_0			T_1	
	ρ_{BCP}	$\nabla^2\rho_{BCP}$	E_{BCP}	ρ_{BCP}	$\nabla^2\rho_{BCP}$	E_{BCP}
Pd73-C59	0.140	0.203	-0.065	0.139	0.151	-0.066
Pd73-N71	0.080	0.344	-0.018	0.081	0.333	-0.019
Pd73-C79	0.136	0.229	-0.061	0.137	0.173	-0.064
Pd73-N93	0.058	0.245	-0.009	0.061	0.249	-0.011

Table S5. Laplacian bond orders of Pt-N and Pt-C bonds of **Pd-1D** and **Pd-1T**.

	**Pd-1D**			**Pd-1T**	
	S_0	T_1		S_0	T_1
Pd21-C7	0.58	0.53	Pd73-C59	0.58	0.52
Pd21-N19	0.25	0.26	Pd73-N71	0.25	0.25
Pd21-C27	0.59	0.54	Pd73-C79	0.59	0.53
Pd21-N41	0.13	0.13	Pd73-N93	0.13	0.14

Table S6. Bond dissociation energies of ligand dissociation for the **Ni-1**, **Pd-1**, **Pt-1**, **Pt-2** and **Pt-3**, respectively. The unit is eV.

	S_0	T_1	S_1	+	-
**Ni-1**	10.10	8.67	7.69	3.79	10.80
**Pd-1**	7.97	5.25	5.19	1.42	8.67
**Pt-1**	10.66	8.27	8.19	4.28	11.43
**Pt-2**	11.91	9.22	9.42	5.90	12.30
**Pt-3**	12.04	9.37	9.42	6.063	12.57

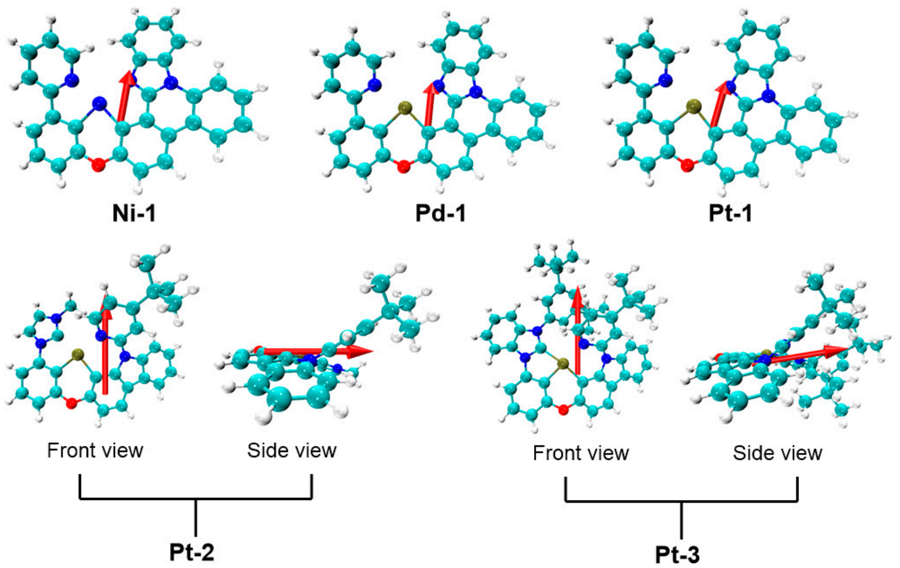

Figure S29. Simulated dipole moment vectors for the **Ni-1**, **Pd-1**, **Pt-1**, **Pt-2** and **Pt-3**, respectively.

The Methodology for intersystem crossing rate

Based on the time-dependent perturbation theory and Born-Oppenheimer adiabatic approximation, the thermal average rate constant from the initial electronic state i with the vibrational quantum numbers v to the final electronic state f with the vibrational quantum numbers u reads:

$$k_{f\leftarrow i} = \frac{2\pi}{\boldsymbol{h}} \sum_{v_i,v_f} P_{iv_i} \left| H'_{fv_f,iv_i} + \sum_{n,v_n} \frac{H'_{fv_f,nv_n} H'_{nv_n,iv_i}}{E_{iv_i} - E_{nv_n}} \right|^2 \delta\left(E_{iv_i} - E_{fv_f}\right)$$

H' denotes the interaction between two different Born-Oppenheimer states, consisting of two contributions:

$$\hat{H}'\Psi_{iv_i} = \hat{H}^{BO}\Phi_i(\mathbf{r};\mathbf{Q})\Theta_{iv_i}(\mathbf{Q}) + \hat{H}^{SO}\Phi_i(\mathbf{r};\mathbf{Q})\Theta_{iv_i}(\mathbf{Q})$$

$\hat{H}^{BO}$ is non-adiabatic coupling and $\hat{H}^{SO}$ is spin-orbital coupling. The **r** and **Q** are the electronic and normal mode coordinates, respectively.

For the organometallic complex, the spin-orbit coupling elements (>100 cm^{-1}) are much larger than those in pure organic fluorescent molecules (<1.0 cm^{-1}). Therefore, it is reasonable to calculate the non-radiative rate constant (k_{nr}) by using the first-order perturbation rate formula. The intersystem crossing rate constant between two electronic states with different spin state can be expressed as,

$$k_{isc}(\omega,T) = \frac{1}{\hbar}\left|\langle\Phi_f|\hat{H}^{SO}|\Phi_i\rangle\right|^2 \int_{-\infty}^{\infty} dt e^{i\omega_n t}[Z_i^{-1}\rho_{ISC}(t,T)]$$

The equation can be written as,

$$k_{isc} = \frac{1}{\boldsymbol{h}^2}|H^{SO}|^2 \int_{-\infty}^{\infty} dt e^{i\omega_{if} t}\left[Z_i^{-1}\rho_{fi}^{(0)}(t)\right]$$

Where the correlation function $\rho_{fi}^{(0)}(t)$ is

$$\rho_{fi}^{(0)}(t) = Tr\left[e^{-i\tau_f\hat{H}^f}e^{-i\tau_i\hat{H}^i}\right] = \sqrt{\frac{det[\boldsymbol{a_f a_i}]}{\det[\boldsymbol{K}]}} \exp\left\{\frac{i}{h}\left[-\frac{1}{2}\underline{F}^T\boldsymbol{K}^{-1}\underline{F} + \underline{D}^T\boldsymbol{E}\underline{D}\right]\right\}$$

Therein

$\tau_f = t/\boldsymbol{h}$

$\tau_i = -t/\boldsymbol{h} - i\beta$

$$a_{i/f,k}(\tau_{i/f}) = \frac{\omega_{i/f,k}}{\sin(\boldsymbol{h}\omega_{i/f,k}\tau_{i/f})}$$

$$b(\tau_{i/f}) = \frac{\omega_{i/f,k}}{\tan(\boldsymbol{h}\omega_{i/f,k}\tau_{i/f})}$$

$$\boldsymbol{A} = \boldsymbol{a_f} + \boldsymbol{S^T a_i S}$$

$$\boldsymbol{B} = \boldsymbol{b_f} + \boldsymbol{S^T b_i S}$$

$$\boldsymbol{E} = \boldsymbol{b_i} - \boldsymbol{a_i}$$

$$\underline{F}^T = \begin{bmatrix} \underline{D}^T \boldsymbol{ES} \\ \underline{D}^T \boldsymbol{ES} \end{bmatrix}$$

$$\underline{F} = \begin{bmatrix} S^T \boldsymbol{E}\underline{D} \\ \boldsymbol{S^T E}\underline{D} \end{bmatrix}$$

$$\boldsymbol{K} = \begin{bmatrix} \boldsymbol{B} & -\boldsymbol{A} \\ -\boldsymbol{A} & \boldsymbol{B} \end{bmatrix}$$

In displaced oscillator model,

$$\boldsymbol{S} = \boldsymbol{I}$$

$$\omega_{i,k} = \omega_{f,k}$$

Then

$$b_{f,k}(\tau_f) - a_{f,k}(\tau_f) = -\omega_k tan\frac{\boldsymbol{h}\omega_k\tau_f}{2}$$

$$b_{f,k}(\tau_f) + a_{f,k}(\tau_f) = \omega_k cot\frac{\boldsymbol{h}\omega_k\tau_f}{2}$$

$$b_{i,k}(\tau_i) - a_{i,k}(\tau_i) = -\omega_k tan\frac{\boldsymbol{h}\omega_k\tau_i}{2}$$

And

$$\frac{i}{\boldsymbol{h}}\left[-\frac{1}{2}\underline{F}^T\boldsymbol{K}^{-1}\underline{F} + \underline{D}^T\boldsymbol{E}\underline{D}\right] = -\sum_{\boldsymbol{k}} S_K\left[(2\bar{n}_k + 1) - (2\bar{n}_k + 1)e^{-i\omega_k t} - \bar{n}_k e^{-i\omega_k t}\right]$$

$$\frac{1}{z_i}\sqrt{\frac{det[\boldsymbol{a_g}]det[\boldsymbol{a_e}]}{\det[\boldsymbol{K}]}} = 1$$

Therein, $\bar{n}_k \equiv \frac{1}{e^{\boldsymbol{h}\omega_k/k_BT}-1}$ is the average number of the photons.

Hence,

$$k_{isc} = \frac{1}{\boldsymbol{h}^2}|H^{SO}|^2 \int_{-\infty}^{\infty} dtF(t)$$

Here

$$F(t) \equiv \exp\left\{ i\omega_{if}t - \sum_{\boldsymbol{k}} S_K\left[(2\bar{n}_k + 1) - (2\bar{n}_k + 1)e^{-i\omega_k t} - \bar{n}_k e^{-i\omega_k t}\right]\right\}$$

The real part of the function F(t) is even, and the imaginary part is odd. The integral is determined by the real part.

$$k_{isc} = \frac{1}{\boldsymbol{h}^2} |H^{SO}|^2 \int_{-\infty}^{\infty} dt\exp \left\{ i\omega_{if} t - \sum_k S_K \left[(2\bar{n}_k + 1) - (2\bar{n}_k + 1) e^{-i\omega_k t} - \bar{n}_k e^{-i\omega_k t} \right] \right\}$$

If $\sum_k S_K \gg 1$, and the short time approximation is adopted

$$e^{\pm i\omega_k t} \approx 1 + i\omega_k t - \frac{1}{2} {\omega_k}^2 t^2$$

The analytic solution can be obtained

$$k_{isc} = \frac{1}{\boldsymbol{h}^2} |H^{SO}|^2 \sqrt{\frac{2\pi}{\sum_k S_k \omega_k^2 (2\bar{n}_k + 1)}} exp \left\{ - \frac{\left(\omega_{fi} + \sum_k S_k \omega_k \right)^2}{2 \sum_k S_K \omega_k^2 (2\bar{n}_k + 1)} \right\}$$

The frequency is replaced by the energy,

$$\bar{E}_k \equiv \left(\bar{n}_k + \frac{1}{2} \right) \boldsymbol{h} \omega_k$$

The final form is obtained

$$k_{isc}(E_{if}) = \frac{1}{\boldsymbol{h}} |H^{SO}|^2 \sqrt{\frac{\pi}{\sum_k \lambda_k \bar{E}_k}} exp \left\{ - \frac{\left(E_{fi} - \sum_k \lambda_k \right)^2}{4 \sum_k \lambda_k \bar{E}_k} \right\}$$

where E_{fi} is the adiabatic excitation energy; $\lambda_k = S_k \hbar \omega_k = \omega_k^2 D_k^2 / 2$ is the reorganization energy for the kth mode; S_k and D_k are the Huang-Rhys factor and displacement of the corresponding mode with frequency ω_k, respectively; As seen from equation, adiabatic excitation energy, reorganization energy and spin–orbit coupling between T_1 and S_0 are three key parameters to govern the non-radiative rate constant.

The non-radiative decay rates equation is solved in MOMAP program, which is developed by Shuai's group.
